# An image processing tool for the detection of anthracycline-induced cardiotoxicity by evaluating the myocardial metabolic activity in [^18^F]FDG PET/CT

**DOI:** 10.1007/s11548-021-02508-9

**Published:** 2021-10-26

**Authors:** Alexander P. Seiffert, Adolfo Gómez-Grande, Gonzalo Castro-Leal, Antonia Rodríguez, David Palomino-Fernández, Enrique J. Gómez, Patricia Sánchez-González, Héctor Bueno

**Affiliations:** 1 Biomedical Engineering and Telemedicine Centre, ETSI Telecomunicación, Center for Biomedical Technology, Universidad Politécnica de Madrid, Madrid, Spain; 2 Department of Nuclear Medicine, Hospital Universitario 12 de Octubre, Madrid, Spain; 3 Department of Hematology, Hospital Universitario 12 de Octubre, Madrid, Spain; 4 Centro de Investigación Biomédica en Red de Bioingeniería, Biomateriales y Nanomedicina (CIBER-BBN), Madrid, Spain; 5 Cardiology Department and Instituto de Investigación Sanitaria (imas12), Hospital Universitario 12 de Octubre, Madrid, Spain; 6 Centro Nacional de Investigaciones Cardiovasculares (CNIC), Madrid, Spain; 7 Facultad de Medicina, Universidad Complutense de Madrid, Madrid, Spain; 8 Centro de Investigación Biomédica en Red de enfermedades Cardiovasculares (CIBERCV), Madrid, Spain; 9 Centro de Investigación Biomédica en Red de Bioingeniería Biomateriales y Nanomedicina (CIBER-BBN), Madrid, Spain

**Keywords:** [^18^F]FDG PET/CT, Cardiotoxicity, Myocardial metabolism quantification, Computer-aided diagnosis

## Abstract

**Purpose:**

Chemotherapy-induced cardiotoxicity is one of the main complications during and after cancer treatment. While echocardiography is the most used technique in clinical practice to evaluate left ventricular (LV) dysfunction, a multimodal approach is preferred for the early detection of anthracycline-induced cardiotoxicity. In this paper, an image processing tool allowing the qualitative and quantitative analysis of myocardial metabolic activity by [^18^F]fluorodeoxyglucose (FDG) positron emission tomography computed tomography (PET/CT) images, acquired routinely during and after cancer treatment, is presented.

**Methods:**

The methodology is based on cardiac single photon emission computed tomography image processing protocols used in clinical practice. LV polar maps are created, and quantitative regional values are calculated. The tool was validated in a study group of 24 patients with Hodgkin or non-Hodgkin lymphoma (HL and NHL, respectively) treated with anthracyclines. Staging, interim and end-of-treatment [^18^F]FDG PET/CT images were acquired and the presented tool was used to extract the quantitative metrics of LV metabolic activity.

**Results:**

Results show an overall increase of metabolic activity in the interim PET image acquired while on treatment compared to staging PET, which then decreased in the end-of-treatment scan. Positive correlation coefficients between staging and interim scans, and negative correlation coefficients between interim and end-of-treatment scans also support this finding. Metabolic changes occur predominantly in the septal region.

**Conclusion:**

The proposed methodology and presented software solution provides the capability to assess quantitatively myocardial metabolism acquired by routine [^18^F]FDG PET/CT scanning during cancer treatment for evaluating anthracycline-induced cardiotoxicity. The [^18^F]FDG PET/CT septal-lateral uptake ratio is proposed as a new quantitative measure of myocardial metabolism.

**Supplementary Information:**

The online version contains supplementary material available at 10.1007/s11548-021-02508-9

## Introduction

The improvement of early detection and cancer therapies, including chemotherapy, has led an increase in survival rates in patients with malignancy in recent years [1]. However, cardiac toxicity is a major secondary effect of several chemotherapy treatments, which may lead to an increased risk of left ventricular systolic dysfunction and heart failure [2] or to other clinical presentations, such as arrythmias and ischemia [3]. Actually cardiovascular (CV) disease is one of the main causes of long-term morbidity and mortality among cancer patients [2].

The prevention, early diagnosis and prediction of cardiotoxicity is defined as a clinical priority by clinical practice guidelines from different cardiology and oncology societies [4–6]. The detection and monitoring of cardiotoxicity is performed essentially by the assessment of left ventricular ejection fraction (LVEF) using echocardiography. The general criteria used to determine cardiotoxicity is a decrease of the LVEF by 5% dropping below 53%, or a decrease by 10% [7, 8]. However, the variability of LVEF measurements with 2D echocardiography may be as high as 10% [9], making it difficult to detect the small changes required for the early detection of cardiotoxicity [10]. One other echocardiographic parameter used to predict cardiac toxicity is global longitudinal strain (GLS). However, the specificity and predictive value of this measure is limited [11, 12]. Late survivors of cancer frequently show abnormalities in global, radial, circumferential and longitudinal strain despite normal LVEF and it is a stronger and more sensitive predictor of cardiotoxicity [8, 13]. Therefore, a multimodal approach for the detection of cardiotoxicity during and after cancer treatment is recommended [14, 15].

The variability and sensitivity in the cardiac evaluation made by echocardiography has led to an increase in the use of other techniques to assess LV function, such as positron emission tomography computed tomography (PET/CT) imaging with [^18^F]fluorodeoxyglucose (FDG). [^18^F]FDG PET/CT allows the *in-vivo* assessment of myocardial glucose metabolism and, potentially, an earlier identification of cardiac toxicity, as it is preceded by myocardial metabolic alterations [16]. Actually, previous studies have suggested that an increase in myocardial radiotracer uptake may be related to anthracycline-induced cardiotoxicity [16–19]. However, as myocardial glucose uptake depends on several other factors, such as diet, blood glucose level, blood insulin level, age, fasting state and drugs [20–22], the substrate that myocardial cells use for their metabolism may change within specific times.

Several studies demonstrated that qualitative and quantitative myocardial uptake patterns can be defined as physiological and pathological [23, 24]. Abnormal uptake patterns have been described for several diseases but are generally identified as an increase in the right ventricular and/or atrial uptake, as well as in excessive uptake within the lateral wall of the LV compared to the septum. Moreover, these studies indicate that, in average, healthy subjects show a 20% decreased uptake in the septum compared to the LV lateral wall, suggesting that a decrease < 20% could be associated with cardiac diseases, such as LV dysfunction.

In this study, an image processing tool is proposed for the qualitative and quantitative analysis of myocardial uptake in [^18^F]FDG PET/CT scans acquired during clinical monitoring of patients on anthracycline chemotherapy. Quantitative parameters that describe the myocardial metabolism are defined. The tool is validated in a study sample of patients with Hodgkin and Non-Hodgkin lymphoma (HL and NHL, respectively) treated with anthracyclines, which are related to anthracycline-induced cardiotoxicity [19, 25, 26].

## Material and methods

### Patients

All patients diagnosed with HL and NHL at *Hospital Universitario 12 de Octubre*, Madrid, Spain, who received chemotherapy between years 2016 and 2019 were considered for this retrospective study. Exclusion criteria were:

- Lack of availability of at least one of the staging, interim and the end-of-treatment (EOT) [^18^F]FDG PET/CT scans.
- Myocardial uptake suppressed in any of the scans.
- Chemotherapy regimen not including anthracyclines.

### Image acquisition

All patients fasted for 6 h previously to the radiopharmaceutical injection and were advised to drink water abundantly and to urinate before the image acquisition. All scans were performed using a Siemens Biograph 6 True-Point PET/CT scanner (Siemens Healthineers AG, Erlangen, Germany). Images were acquired 45–90 min after the intravenous injection of an average dose of 4.89 MBq/kg of [^18^F]FDG. The scan duration per bed was 3 min and the number of beds depended on the patient height. Images were reconstructed with a point spread function (PSF) algorithm (3 iterations, 21 subsets, all-pass filter), and scatter and random correction were performed. The reconstructed PET images had a matrix size of 168 × 168 and voxel size of 4.0728 × 4.0728 mm. The slice thickness was 5 mm. A low-dose CT scan with matrix size of 512 × 512 and voxel size 0.9766 × 0.9766 mm was also acquired for attenuation correction. Median time between staging and interim scans was 91 days (range: 50–272) and median time between interim and EOT scans was 81 days (range: 57–224).

### Image processing

First, PET images are filtered to reduce the Poisson noise and enhance the signal to noise ratio to improve the LV boundary delimitation. The used filter was designed by [27] (http://www.cs.tut.fi/~foi/invansc/). Then, a preliminary localization of the LV in the PET image is performed based on the CT scan [28]. The localization obtained from the CT processing is not totally accurate and requires manual correction. Once it is localized, a volume of interest (VOI) containing the LV is segmented. In order to avoid possible tumor masses close to the heart, an ellipsoid is used since it represents better the shape of the LV.

**Fig. 1:**
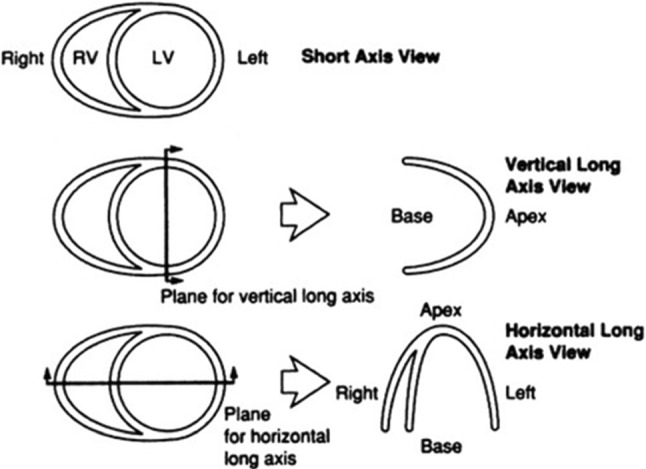
Schematic representation of ventricular axes. Adapted from [29]

For quantitative analysis of the LV, a standardized uptake values (SUV) map of the VOI is calculated. The LV is oriented along the standardized heart axes shown in Fig. 1. These axes are the horizontal long axis (HLA), vertical long axis (VLA), and short axis (SA). To accomplish the standard orientation the LV must be rotated twice, first through the HLA until the septum is vertical in the image plane, and then through the VLA so the inferior wall is horizontal [29].

**Fig. 2:**
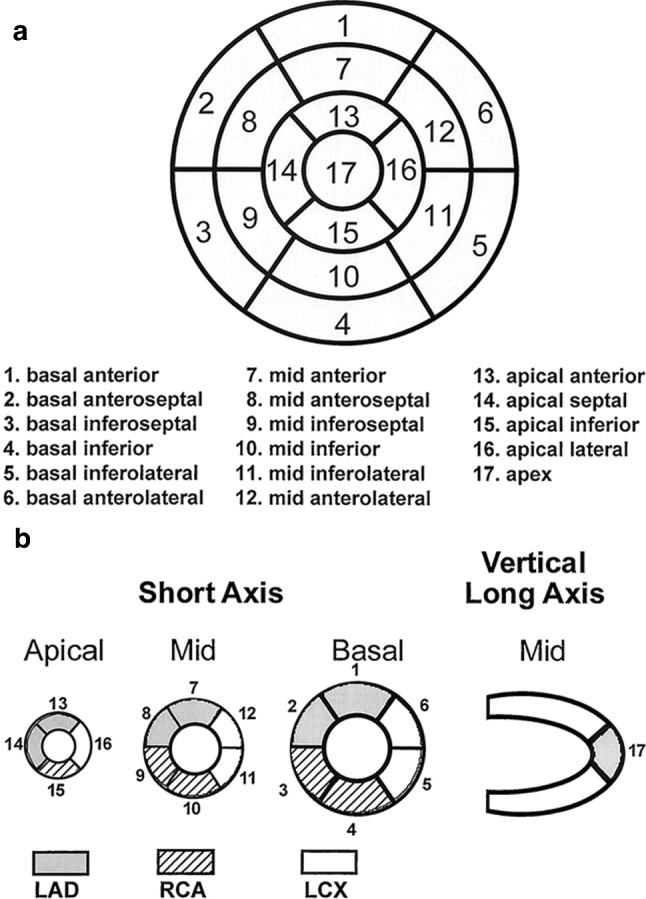
**a** Segments of the polar map according to the AHA-17 criteria with its corresponding names. **b** Mapping of the polar map segments to the three vascular territories: Left Anterior Descending artery (LAD), Right Coronary Artery (RCA), Left CircunfleX artery (LCX). Adapted from [29]

Once the LV is oriented correctly, the myocardium wall within the VOI is defined and localized, forming a ring of pixels in the SA view. The pixels that form the ring are obtained as the ones with maximum SUV in each direction, from 0º to 359º with 1º increments, starting from the center. These rings are generated iteratively for each axial slice and the center is recalculated for each iteration. Then, a polar map is created that represents the uptake pattern of the myocardium. It is created based on the University of Michigan standard [30], which assumes that the lateral and septal basal limits are equal, and that the first and last myocardium rings are those with nonzero elements. The different regions and its localization in the polar map are shown in Fig. 2.

Lastly, the metabolic activity of the different myocardial regions is quantified by extracting SUVmax and SUVmean from each segment of the polar map. For statistical analysis, the weighted average SUVmean of all segments except the apex is calculated, as well as the SUVmax of the septal (segments 2, 3, 8 and 9) and lateral (segments 5, 6, 11 and 12) regions. Regarding the artery territories, the ratio between the left anterior descending artery (LAD) and the left circumflex artery (LCX), as well as the ratio between the right coronary artery (RCA) and the LCX are calculated. Lastly, a septal-lateral uptake ratio (SLUR) is calculated based on the SUVmean of the corresponding segments of the polar map.

### Statistical analysis

Quantitative variables are represented as mean ± standard deviation. Statistical analysis was performed separating the patients according to the chemotherapy regimen, as well as for a combined group. Differences of SUVmax, SUVmean, artery ratios and SLUR between staging, interim and EOT scans were studied with Friedman’s test and *post-hoc* pair-wise tests with Bonferroni adjusted α values. Moreover, pair-wise linear correlation between quantitative measurements was studied by calculating the point-biserial correlation coefficient *r* to evaluate whether the overall changes between scans are positive or negative. *P*-values < 0.05 were considered statistically significant and statistical analyses were performed in SPSS software version 19.00 (IBM Corp., Armonk, NY).

## Results

### Study group

**Fig. 3:**
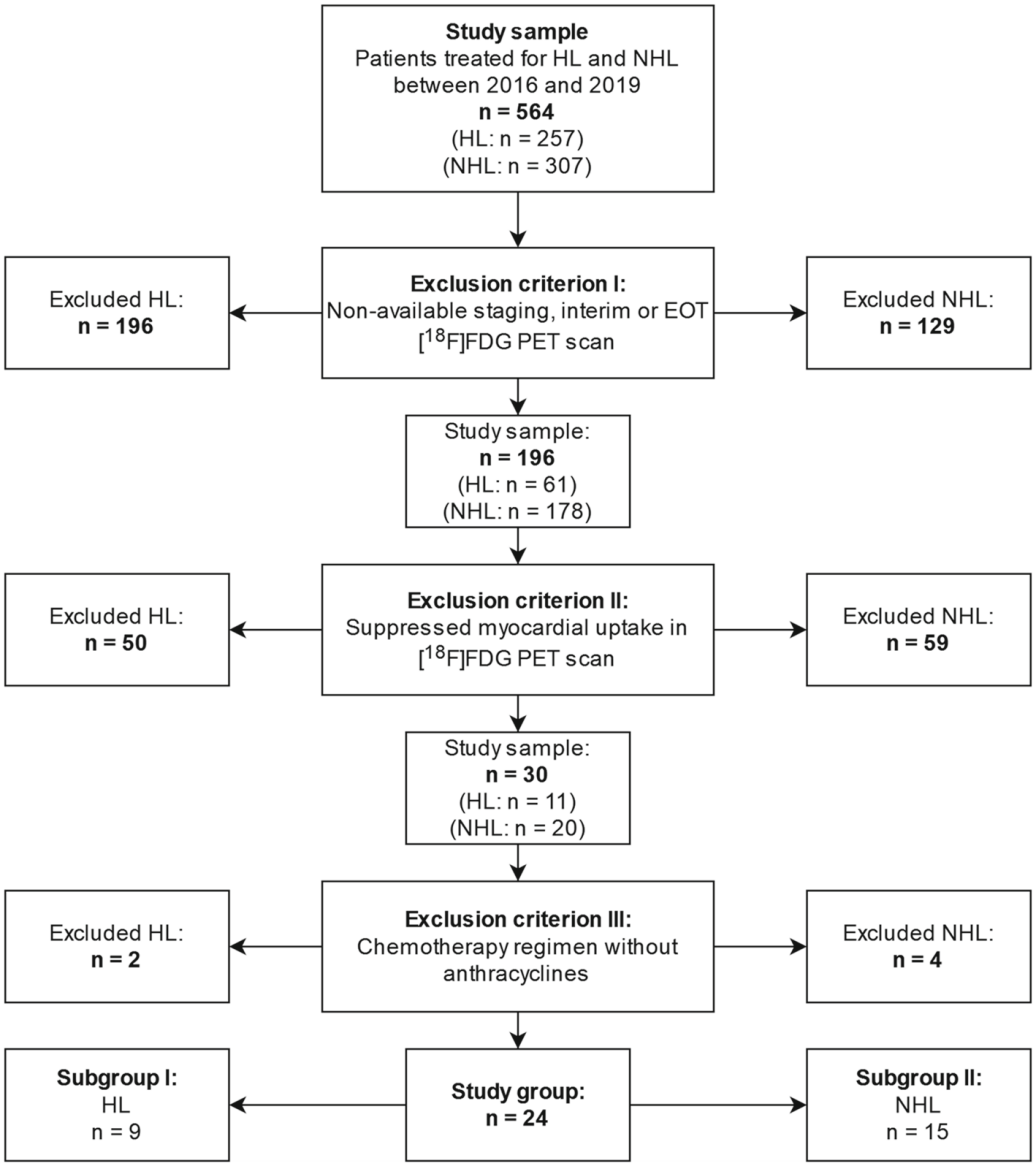
Study group selection flow graph (*n* = 24)

The initial study group consisted of 564 patients with lymphoma, who were divided into two groups, those with HL (*n* = 257) and patients with NHL (*n* = 307). Of these, 196 patients with HL and 129 patients with NHL had at least one staging, interim or EOT scan not available and were excluded. There was no myocardial uptake in at least one of the [^18^F]FDG PET/CT scans in 50 HL patients and in 59 NHL patients. Lastly, chemotherapy was not based on anthracyclines in 2 patients with HL and 4 patients with NHL. One additional case had an EOT scan with low septal uptake, indistinguishable from the blood pool and surrounding tissue and, therefore, could not be processed. The final study group included 9 HL patients and 15 NHL patients (Fig. 3).

Demographics and clinical characteristics of the study group are shown in Table 1. Five patients from each group presented at least one cardiovascular risk factor, being smoking (active or not) the most frequent overall (8 patients). Chemotherapy regimens were ABVD (adriamycin, bleomycin sulfate, vinblastine sulfate, dacarbazine) for HL patients and R-CHOP (rituximab, cyclophosphamid, hydroxydaunorubicin, oncovin, prednisone) and its variants for NHL patients. No patient showed abnormal echocardiography data, i.e. a baseline left ventricular ejection fraction (LVEF) < 55%, regional wall motion abnormalities at rest, myocardial hypertrophy (any LV segment ≥ 13 mm), moderate or severe valvular heart disease, prior history of cardiotoxicity or radiotherapy and inadequate acoustic window [31]. Patients did not receive radiotherapy in the time between baseline and EOT [^18^F]FDG PET images or at the moment of image acquisition that could impact myocardial [^18^F]FDG uptake.

**Table 1:** Patient demographics and clinical data

	HL (*n* = 9)	NHL (*n* = 15)
*Sex*		
Male	2	8
Female	7	7
Age (years)		
Mean ± SD	30.1 ± 8.7	48.7 ± 11.4
Range	21–49	35–72
*Clinical data*		
Cardiovascular risk factors	5	5
Smoker	1	4
Ex-smoker	2	1
Obesity	1	1
Diabetes	1	1
*Chemotherapy regimen*		
R-CHOP	0	10
R-CHOP variant	0	5
ABVD	9	0
*Echocardiography data*		
Normal LVEF	9	15
Abnormal	0	0

### Implementation

The proposed image-processing tool is implemented in MATLAB R2019a. As mentioned above, manual corrections are needed at several certain steps. Therefore, a graphical user interface is developed based on MATLAB’s GUIDE (Graphical User Interface Development Environment). Individual tabs, henceforth called modules, are dedicated to each distinct step, namely segmentation, orientation, and the results.

**Fig. 4:**
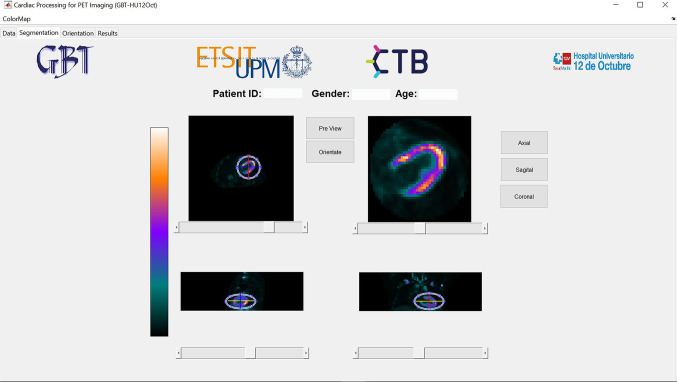
Segmentation module showing the localized and segmented left ventricle

The loaded [^18^F]FDG PET/CT scan is displayed in the segmentation module as seen in Fig. 4 after filtering and LV localization. Three ellipses, one for each image plane, are used to define the ellipsoid containing the LV VOI. The resulting VOI can be pre-visualized to ensure that the LV is completely segmented before going to the next step.

**Fig. 5:**
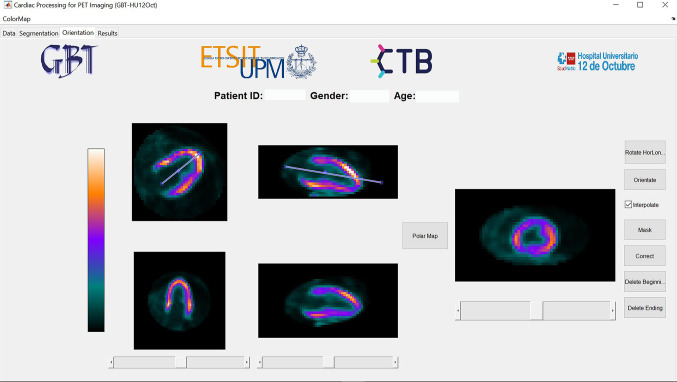
Orientation module showing the auxiliary lines and a correctly oriented left ventricle

The orientation of the LV and its three axes (HLA, VLA, SA) is performed in two steps using auxiliary lines (see Fig. 5). First, the HLA is oriented with the auxiliary line and its rotation angle is used to rotate the image. For a correct orientation, it needs to be positioned parallel to the septum. The result is displayed underneath after pressing the ‘RotateHorLonAx’ button. Then, the VLA is oriented, which needs to be parallel to the inferior wall. The button ‘Orientate’ can be pressed when the VLA is oriented and the result is shown underneath, as well as the sagittal plane of the LV on the rightmost image.

Once the LV is oriented correctly, the data for the creation of the polar map is obtained based on the axial slides. First, a binary mask approximating the myocardium is generated based on the Otsu’s method. Then, the algorithm searches for the points of maximum intensity and creates the necessary rings. However, a correction might be needed when the rings are not closed or include redundant pixels. Three scenarios require a correction: (1) tumoral cells near the myocardium, (2) the uptake level of the myocardium is low, and Otsu’s method is not able to distinguish it from the blood pool or surrounding tissue, and (3) the papillary muscles are segmented. With the buttons on the right below the ‘Interpolate’ checkbox, the user can redraw the mask that approximately segments the myocardium before generating the rings for the polar map.

**Fig. 6:**
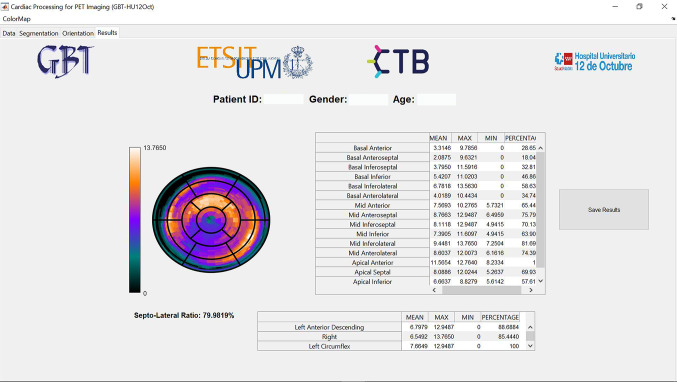
Results module showing the polar map, segmental and regional quantitative parameters

Once the image processing is complete, the polar map is displayed in the ‘Results’ module (see Fig. 6). Moreover, quantitative metrics are extracted and displayed in two tables, one for the segmental values and another one for the vascular territories. The data can then be saved in the database and accessed again by the user.

### Validation results

Table 2 shows the average LV uptake over the three scans. An overall increase of the myocardial uptake can be observed on the interim scans compared to the staging and EOT evaluations, and the latter showing the lowest uptake, except in the patients treated with the ABVD regimen. Moreover, the uptake is higher in the lateral region than the septal region. Regarding the SLUR, the same pattern can be observed but the lowest ratio usually appears at the EOT scan. The only statistically significant differences between quantitative parameters were obtained for the LAD/LCX ratio in the R-CHOP group (*p* = 0.041). However, *post-hoc* analyses showed no statistically significant differences.

**Table 2:** Quantitative metrics of myocardial uptake

Chemotherapy regimen	Staging scan			Interim scan			EOT scan			*P*-value		
	R-CHOP	ABVD	Both	R-CHOP	ABVD	Both	R-CHOP	ABVD	Both	R-CHOP	ABVD	Both
SUVmean	6.77 ± 5.04	5.43 ± 2.68	6.27 ± 4.29	8.44 ± 4.46	6.65 ± 3.26	7.77 ± 4.07	7.03 ± 4.13	7.58 ± 4.88	7.24 ± 4.33	0.627	0.895	0.607
*SUVmax*												
Septal Region	9.47 ± 6.76	8.15 ± 4.00	8.97 ± 5.81	11.35 ± 5.67	9.32 ± 3.69	10.59 ± 5.24	10.05 ± 5.94	10.44 ± 7.29	10.20 ± 6.32	0.627	0.717	0.453
Lateral Region	10.86 ± 7.30	9.43 ± 5.27	10.32 ± 6.53	13.39 ± 6.44	10.41 ± 4.48	12.27 ± 5.86	11.96 ± 5.99	12.67 ± 7.79	12.23 ± 6.57	0.766	0.895	0.747
LAD/LCX	81.73% ± 7.99	85.78% ± 8.14	83.25% ± 8.12	84.30% ± 6.05	87.78% ± 4.11	85.61% ± 5.58	78.75% ± 7.94	84.43% ± 10.37	80.88% ± 9.15	**0.041**	0.459	0.167
RCA/LCX	81.62% ± 10.01	84.67% ± 4.82	82.77% ± 8.44	82.33% ± 7.54	86.84% ± 6.59	84.02% ± 7.39	78.80% ± 11.77	81.93% ± 8.67	79.97% ± 10.62	0.819	0.717	0.607
SLUR	80.28% ± 11.09	83.95% ± 5.94	81.66% ± 9.51	83.65% ± 8.22	86.38% ± 7.54	84.67% ± 7.92	78.81% ± 12.13	81.23% ± 10.14	79.72% ± 11.26	0.247	0.895	0.582

Statistically significant correlation coefficients are given in bold

Table 3 shows the correlation coefficients of the quantitative metrics between the staging and interim scans, as well as between the interim and EOT scans. A statistically significant negative correlation can be observed for the LAD/LCX ratio between the interim and EOT scans when analyzing the R-CHOP group (*r* =  − 0.3706, *p* = 0.0401) and both groups simultaneously (*r* = − 0.3004, *p* = 0.036).

**Table 3:** Correlation coefficients of quantitative metrics between staging and interim scans, and interim and EOT scans

Chemotherapy regimen		*r* (*p*-value): Staging scan—Interim scan			*r* (*p*-value): Interim scan—EOT scan		
		R-CHOP	ABVD	Both	R-CHOP	ABVD	Both
SUVmean		0.1756 (0.3451)	0.2057 (0.3992)	0.1783 (0.2204)	− 0.1639 (0.3787)	0.1144 (0.6418)	− 0.0635 (0.6648)
*SUVmax*							
Septal region		0.1488 (0.4246)	0.1546 (0.5284)	0.1461 (0.3165)	− 0.1107 (0.5537)	0.0999 (0.6849)	− 0.0341 (0.8160)
Lateral region		0.1837 (0.3229)	0.1029 (0.6760)	0.1568 (0.2822)	− 0.1162 (0.5341)	0.1801 (0.4616)	− 0.0038 (0.9794)
LAD/LCX		0.1819 (0.3278)	0.1580 (0.5193)	0.1689 (0.2461)	**− 0.3706 (0.0401)**	− 0.2135 (0.3810)	**− 0.3004 (0.0360)**
RCA/LCX		0.0405 (0.8287)	0.1895 (0.4380)	0.0795 (0.5871)	− 0.1788 (0.3361)	− 0.3114 (0.1948)	− 0.2181 (0.1323)
SLUR		0.1728 (0.3530)	0.1814 (0.4583)	0.1716 (0.2386)	− 0.2310 (0.2114)	− 0.2839 (0.2395)	− 0.2490 (0.0845)

Statistically significant correlation coefficients are given in bold

## Discussion

[^18^F]FDG PET/CT scans routinely acquired for monitoring chemotherapy response in cancer patients could yield valuable information for predicting and diagnosing anthracycline-induced cardiotoxicity. The present work presents a novel image-processing algorithm to extract quantitative metrics that describe the myocardial metabolic activity of the LV. The developed tool offers polar maps for qualitative analysis as well as segmental SUVmean and SUVmax values. A ratio between septal and lateral uptake is also calculated. Cardiac polar maps are useful tool for the evaluation of cardiac abnormalities. Usually, these are visually interpreted in clinical settings.

The quantitative metrics showed a tendency to increase from the staging to the EOT scan, which is in line with the findings of other studies [16, 17, 19]. However, the highest values were observed in the interim scan and the values used to drop in the EOT scan. This is also reflected in positive correlation coefficients between the first two scans and negative correlation coefficients between the last two scans. Borde et al. [16] suggested that an increase in the SUV within the myocardium could be related to the activation of a metabolic route previous to myocardial damage. Bauckneht et al. [17] found an increase of LV SUV during and after chemotherapy with doxorubicin compared to baseline. Sarocchi et al. [19] found a relationship between the SUVmax increase and the later drop of the LVEF. Moreover, the value of the SLUR parameter obtained in the present study was generally lower in some EOT scans, which could be related to myocardial damage [13]. These results suggest that there are metabolic changes related to myocardial damage predominantly in the septal region. All the above indicates that the parameters studied might be useful to detect signs of cardiotoxicity in its preliminary phase, while it is easier to be reverted. However, the patterns observed in this study were not statistically significant except in one case. In future works, additional quantitative features will be extracted, with special emphasis on volumetric data and radiomic features, which will be used with techniques like machine learning and artificial intelligence to predict the risk of patients to develop anthracycline-induced cardiotoxicity.

While the methodology follows the steps used for the processing of cardiac SPECT images in clinical practice, a fully automated workflow is preferred. The current implementation requires time to finish a case and especially the segmentation of the LV VOI might be complex without practice. Moreover, the creation and possible correction of the LV mask and creation of the polar map depend on the criteria of the user and increases inter user variability. The visual interpretation of the polar maps is also subject to inter observer variability. Therefore, an automated classification model as proposed by previous studies could be implemented in future versions of the tool [32, 33]. Further validation of the generated polar maps is also required and will be performed based on a blinded multi-reader approach in future studies. Regarding the graphical user interface of the presented tool, it is currently a provisional implementation to provide the necessary functionality to process the images and obtain the quantitative metrics. Future work will be devoted to improve its functions, usability and aesthetics.

On the other hand, the limitations of the study are mainly related to the retrospective nature of the study group without specific anthracycline-induced cardiotoxicity confirmation, which could explain the lack of statistically significant results. Suppressed myocardial uptake in any of the [^18^F]FDG PET/CT scans was considered an exclusion criterion (excluded: HL, *n* = 50 and NHL, *n* = 59). Even though this methodological decision is justifiable for the validation of the proposed tool, further extended versions of the software for research or clinical use should also quantify minimal myocardial FDG uptake. The population of the study is not numerous and lacks a control group to compare the results with. Moreover, patients have not been monitored enough to relate the results obtained with other quantitative metrics like the LVEF or a confirmed diagnosis of anthracycline-induced cardiotoxicity. Finally, some variables such as the diet or the chemotherapy dose could not be controlled in this retrospective study group and which are known to affect the myocardial glucose uptake and the cardiotoxic damage. As stated by Bauckneht et al. [34], prospective studies are necessary to validate the preclinical findings of the usefulness of [^18^F]FDG PET/CT images for their role in cardiotoxicity. Therefore, one of the main goals of our future studies is the validation of the presented methodology in a prospective cohort, as well as in additional retrospective cohorts with healthy control subjects and patients of confirmed cardiotoxicity. Additionally, the proposed methodology and image processing tool is planned to be validated with cohorts comprehending other types of chemotherapy-induced cardiotoxicity. Lastly, this algorithm could also be used for the quantitative analysis of the ventricular metabolic uptake in other pathologies like sarcoidosis.

## Conclusions

Clinical guidelines recommend conducting a [^18^F]FDG PET/CT scan before, during and after chemotherapy treatment. The possibility of assessing quantitatively the myocardial metabolism in scheduled protocol PET/CT scans is an attractive opportunity for the early detection of cardiotoxicity with important clinical implications, including individual tailoring of chemotherapy options to prevent cardiac damage when cardiac damage is reversible. An image processing tool to obtain quantitative metrics of cardiac metabolism from clinical routine [^18^F]FDG PET/CT scans based on existing standards for cardiac segmentation and analysis is presented. The [^18^F]FDG PET/CT septal-lateral uptake ratio may be a new complementary measure to predict anthracycline chemotherapy-related cardiotoxicity.

## Supplementary Information

Below is the link to the electronic supplementary material

Supplementary file1 (DOCX 1816 kb)

## Declarations

### Availability of data and material

The datasets used and/or analysed during the current study are available from the corresponding author on reasonable request.

### Code availability

The code is available from the corresponding author on reasonable request.
