# Supplementary material for: An image processing tool for the detection of anthracycline-induced cardiotoxicity by evaluating the myocardial metabolic activity in [18F]FDG PET/CT
Source: Int J Comput Assist Radiol Surg. 2021 Oct 26;17(2):373–83. doi: 10.1007/s11548-021-02508-9 (PMC8784503; doi:10.1007/s11548-021-02508-9)
Supplement: Supplementary file 1 — Supplementary file1 (DOCX 1816 kb) [file 11548_2021_2508_MOESM1_ESM.docx]

**An image processing tool for the detection of cardiotoxicity by evaluating the myocardial metabolic activity in [^18^F]FDG PET/CT - Supplementary Materials**

Alexander P. Seiffert ^*^, Adolfo Gómez-Grande ^*^, Gonzalo Castro-Leal, Antonia Rodríguez,

David Palomino-Fernández, Enrique J. Gómez, Patricia Sánchez-González, Héctor Bueno

* Alexander P. Seiffert and Adolfo Gómez-Grande contributed equally to this work and are listed as co-first authors.

**1. Supplementary material to section 2. Material and methods**

The polar map computed with the presented image processing tool follows the AHA-17 criteria [1] which states that the basal, mid and apical regions represent 35%, 35% and 30% of the LV, respectively. Moreover, the apex is considered as the first 30% of the apical region.

The SLUR is obtained as the weighed mean uptake of the septum divided by the weighed mean uptake of the lateral wall of the LV. The calculations for the SLUR are based on the AHA-17 [2] standard and the following considerations (see Fig. 1S):

- The apical ring of the polar map represents 70% of the apical region (30% of the LV). Its septal (segment 14) and lateral (segment 16) segments each correspond to 25% of the apical ring.
- The basal and mid rings represent 70% of the LV cavity and the lateral or septal segments (segments 2, 3, 8 and 9, and segments 5, 6, 11 and 12, respectively) correspond to 23.32% of the LV.
- Therefore, the lateral or septal region correspond to 28.57% of the LV.

| 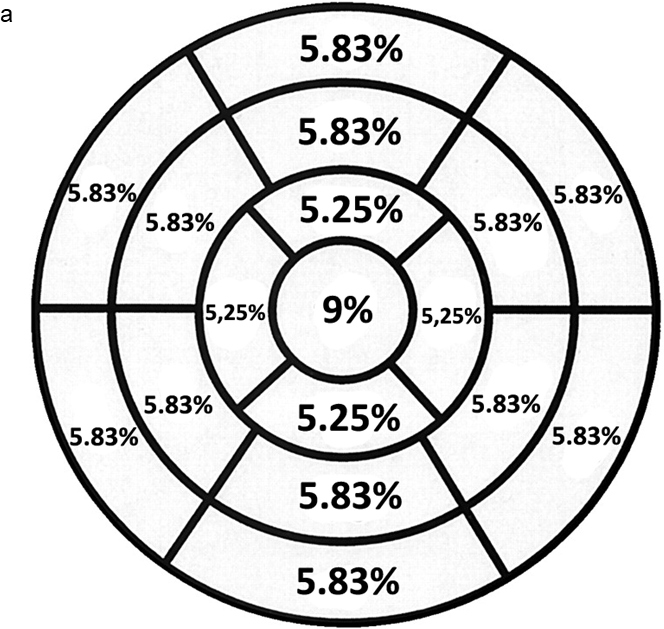 | 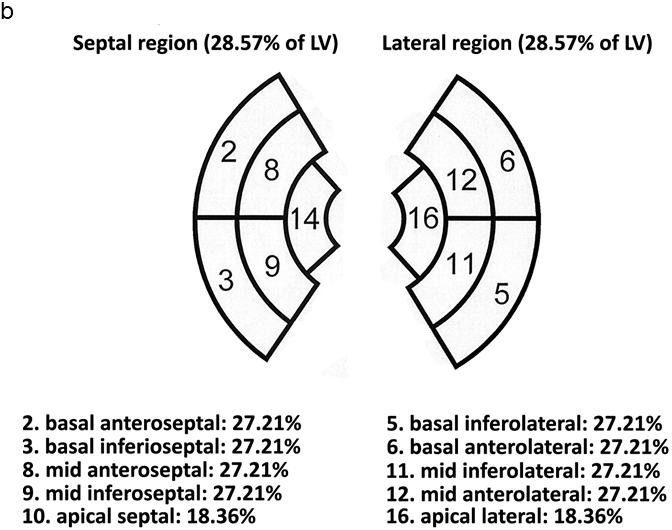 |
| --- | --- |

**Fig. 1S.** a: Weight of each segment of the left ventricle. b: Weight of each segment of the septal (left) and lateral (right) region. Adapted from [1].

Each segment is assigned a percentage corresponding to its weight, as can be seen in Fig. 1S. Moreover, these percentages are then converted to account for the weight of the whole region. The SLUR is then calculated based on Eq. 1:

| $\text{SLUR }\text{ }\text{=}\text{ }\frac{\left[ {\text{(}{SUVmean}_{2}\text{+}{SUVmean}_{3}\text{+}{SUVmean}_{8}\text{+}{SUVmean}_{9}\text{)}}/4 \right]\cdot0.8164\text{+}{SUVmean}_{14}\text{∙0.1836}}{\left[ {\text{(}{SUVmean}_{5}\text{+}{SUVmean}_{6}\text{+}{SUVmean}_{11}\text{+}{SUVmean}_{12}\text{)}}/4 \right]\cdot0.8164\text{+}{SUVmean}_{16}\text{∙0.1836}}$ | **Eq. 1S** |
| --- | --- |
| **2. Supplementary material to section 3. Results**  An example of the obtained polar maps after validating the developed tool with the images of the study group is shown on the bottom rows of Fig. 2S. To assess the correct generation of the maps, they are compared to polar maps obtained with Carimas and PCARDP. As can be observed, the polar maps of the three tools show similar uptake patterns in all regions and are visually comparable. Differences can be observed mainly in slight changes of the orientation, which are due to operator variability. However, no statistical comparison between all three maps has been performed, which is planned for future works.   \| 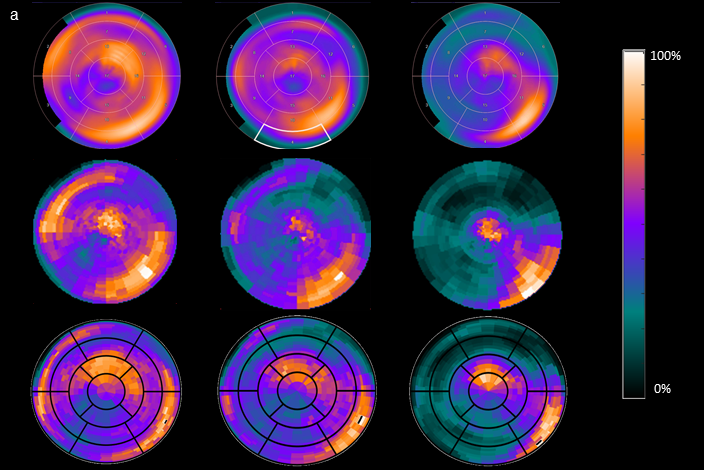 \| 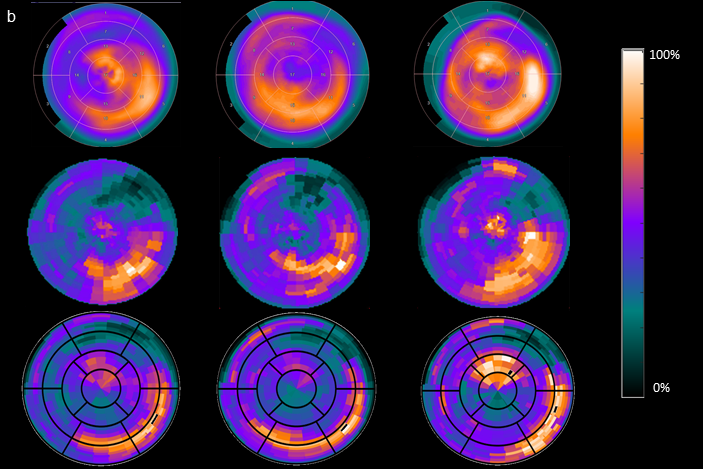 \| \| --- \| --- \| \| 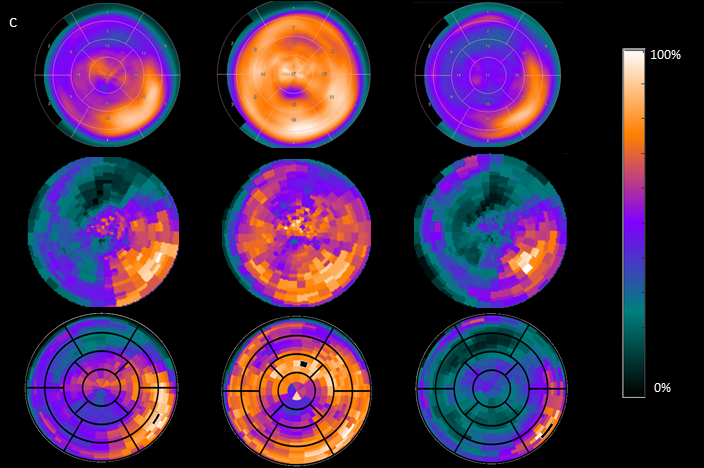 \| \|   **Fig. 2S.** Comparison between polar maps of the staging (left column), interim (central column) and end-of-treatment (right column) [^18^F]FDG PET/CT scans for three cases (a, b and c). Top row: Carimas; middle row: PCARDP; bottom row: the present software.  **References** |  |

1. Cerqueira MD, Weissman NJ, Dilsizian V, et al (2002) Standardized myocardial segmentation and nomenclature for tomographic imaging of the heart: A Statement for Healthcare Professionals from the Cardiac Imaging Committee of the Council on Clinical Cardiology of the American Heart Association. Circulation 105:539–542. https://doi.org/10.1161/hc0402.102975

2. Israel O, Weiler-Sagie M, Rispler S, et al (2007) PET/CT quantitation of the effect of patient-related factors on cardiac 18F-FDG uptake. J Nucl Med 48:234–239
